# The complement system in hypertension and renal damage in the Dahl SS rat

**DOI:** 10.14814/phy2.13655

**Published:** 2018-03-29

**Authors:** Jean F. Regal, Connor F. Laule, Luke McCutcheon, Kate M. Root, Hayley Lund, Shireen Hashmat, David L. Mattson

**Affiliations:** ^1^ Department of Biomedical Sciences University of Minnesota Medical School, Duluth Campus Duluth Minnesota; ^2^ Department of Physiology Medical College of Wisconsin Milwaukee Wisconsin

**Keywords:** C3a, complement, kidney, salt‐sensitive hypertension

## Abstract

Evidence indicates the immune system is important in development of hypertension and kidney disease. In the Dahl Salt‐Sensitive (SS) rat model, lymphocytes play a role in development of hypertension and kidney damage after increased sodium intake. Recent transcriptomic analyses demonstrate upregulation of the innate immune complement system in the kidney of Dahl SS rat fed a high‐salt diet, leading us to hypothesize that inhibition of complement activation would attenuate development of hypertension and kidney damage. Male Dahl SS rats on a low salt (0.4% NaCl) diet were instrumented with telemeters for continuous monitoring of arterial blood pressure. Animals received saline vehicle (Control) or sCR1, a soluble form of endogenous Complement Receptor 1 (CR1; CD35) that inhibits complement activation. At Day 0, rats were switched to high salt (4.0% NaCl) diet and assigned to sCR1 (15 mg/kg per day) or Control groups with daily ip injections either days 1–7 or days 14–18. Urine was collected overnight for determination of albumin excretion. Treatment with sCR1, either immediately after high‐salt diet was initiated, or at days 14–18, did not alter development of hypertension or albuminuria. The sCR1 dose effectively inhibited total hemolytic complement activity as well as C3a generation. High salt caused an increase in message for complement regulator *Cd59*, with minimal change in *Crry* that controls the C3 convertase. Thus, innate immune complement activation in the circulation is not critical for development of hypertension and kidney damage due to increased sodium intake, and therapeutic manipulation of the complement system is not indicated in salt‐sensitive hypertension.

## Introduction

Animal studies and human data indicate that the immune system is important in the development of hypertension and kidney damage (Rodriguez‐Iturbe et al. 2017). Work from our laboratory in the Dahl Salt‐Sensitive (SS) rat, an established model of hypertension, indicates that increasing sodium intake results in increased infiltration of immune cells in the kidney which amplifies the development of hypertension and kidney damage (Mattson et al. 2006; De Miguel et al. 2010, 2011a,b). Moreover, genetic mutation to reduce T lymphocytes (Rudemiller et al. 2014), genetic deletion of T‐ and B‐lymphocytes (Mattson et al. 2013) or chronic immunosuppressive therapy (De Miguel et al. 2010, 2011a,b; Mastellos et al. 2013) in SS rats decreases immune cell infiltration, decreases oxidative stress in the kidney, and attenuates salt‐sensitive hypertension and renal disease. Thus, the role of the adaptive immune system in salt‐sensitive hypertension and renal disease is well established.

The complement system is an innate immune amplification system that operates intracellularly and extracellularly to amplify adaptive immunity as well as work independently of adaptive immunity to ward off infection. Excessive complement system activation can have pathological effects on its own or in concert with humoral or cellular immunity, and the complement system has been implicated in pathophysiology of both hypertension and renal disease. As evidence, the degree of disease in Sprague‐Dawley rats subjected to bilateral ischemia reperfusion injury and high salt is dependent upon infiltrating immune cells in the kidney (Pechman et al. 2008) and these rats also demonstrate increased complement in the kidney (Basile et al. 2005). Increased excretion of complement‐related proteins has been observed in patients with IgA nephropathy, patients with proteinuria, and patients with systemic lupus erythematosus (Ueda et al. 1995; Morita et al. 2000; Onda et al. 2011) as well as in hypertension in pregnancy (Burwick et al. 2013). Plasma levels of complement C3 and C4 have been linked to hypertension and cardiovascular risk in humans (Engstrom et al. 2007; Nilsson et al. 2014). In spontaneously hypertensive rats, C3 is involved in epithelial to mesenchymal cell transitions and activation of the renal renin angiotensin system leading to hypertension and proteinuria (Han et al. 2012; Zhou et al. 2013; Ikeda et al. 2014). In DOCA salt‐induced vascular injury in mice, the complement activation product C5a mediates macrophage polarization and the resultant hypertension‐induced vascular injury and dysfunction (Ruan et al. 2010, 2015). In placental ischemia‐induced hypertension in the pregnant rat, inhibiting complement activation attenuated hypertension providing a mechanistic link between complement activation and hypertension in pregnancy (Lillegard et al. 2013, 2014).

The role of the complement system in salt‐sensitive hypertension and kidney injury is unknown, though two recent transcriptomic analyses have indicated that the complement pathway is upregulated in the kidney of the Dahl SS rat fed a high‐salt diet (Liu et al. 2014; Geurts et al. 2015). We therefore hypothesized that activation of the complement system mediates the secondary development of hypertension and renal disease in the Dahl SS rat. To test this hypothesis, we determined if inhibiting complement activation with soluble complement receptor 1 (sCR1), a soluble form of an endogenous regulator of complement activation, attenuated the development of hypertension and proteinuria in the Dahl SS rat fed a high‐salt diet.

## Materials and Methods

Experiments were performed on age‐matched, male Dahl SS/JrHsdMcwi (SS) rats obtained from inbred colonies at the Medical College of Wisconsin and provided food and water *ad lib*. Breeding stock and study animals were fed purified AIN‐76A rodent chow (Dyets, Inc, Bethlehem, PA) containing 0.4% NaCl from weaning. Experimental animals were fed 0.4% NaCl chow until 9 weeks of age and were then fed the same diet containing 4.0% NaCl for the remainder of the experimental protocol. The Institutional Animal Care and Use Committee of the Medical College of Wisconsin approved all studies.

### Experimental Plan

The influence of an elevated NaCl intake on the development of hypertension and renal damage in Dahl SS rats was assessed in rats randomly assigned to one of the following groups: (1) Dahl SS rats + ip saline vehicle from days 1–7 of high salt, *n* = 6 animals (Control); (2) Dahl SS rats + ip sCR1 (15 mg/kg) from days 1–7 of high salt, *n* = 5 animals (sCR1); (3) Dahl SS rats + ip saline vehicle from days 14–18 of high salt, *n* = 7 animals (Control); (4) Dahl SS rats + ip sCR1 (15 mg/kg) from days 14–18 of high salt, *n* = 5 animals (sCR1). Based upon our previous observations indicating that immune mechanisms amplify salt‐sensitive hypertension in Dahl SS following an initial increase in blood pressure, the present experiments were designed to assess the importance of complement activation in the early (days 1–7 of high salt) and the secondary (days 14–18 of high salt) phase of hypertension.

Seven‐week‐old male rats, maintained on a low salt (0.4% NaCl) diet were instrumented with telemetry transmitters (DSI PA) for the continuous, direct measurement of arterial blood pressure as previously described (Rudemiller et al. 2014, 2015). The blood pressure and albuminuria in female rats exhibits the same pattern but the magnitude of the response is blunted (Mattson et al. 2008). The rats recover from surgery for 5 days and then arterial pressure is measured continuously while the rats are maintained on the low salt (0.4% NaCl) diet. An overnight urine collection assessed baseline proteinuria/albuminuria on low salt at day 0 (7LS). The rat chow is then switched to a high salt content (4.0% NaCl) on Day 0 which results in the development of elevated arterial blood pressure within 2–3 days and the development of proteinuria and albuminuria in 1–2 weeks. An overnight urine collection was then obtained after 7, 14, and 21 days of the high‐salt diet (7HS, 14HS, 21HS). Rats were treated with sCR1 or saline vehicle (Control) on high‐salt days 1–7, to test for the primary/initial effects of complement on the disease phenotype. In these animals, serum was obtained from tail vein on day 8 and at euthanasia from the abdominal aorta on day 21. Animals were euthanized with an overdose of sodium pentobarbital.

In a separate group of animals, rats were treated with sCR1 or saline vehicle (Control) with daily ip injections on days 14–18 to test for the secondary/delayed effects of complement on disease progression. In this group of animals, serum was obtained at euthanasia from the abdominal aorta on day 22. Arterial pressure was also monitored daily during this experiment. Overnight urine collections were done on low‐salt diet as well as on Day 7, 14, and 21 of the high salt diet. Animals were euthanized with an overdose of sodium pentobarbital following 22 days on the high‐salt diet.

### *sCR1*

sCR1 (Celldex Therapeutics, Needham, MA) was provided at 5.4 mg/mL in PBS containing mannitol, and was dialyzed against endotoxin free saline (vehicle) prior to ip injection. sCR1 is a soluble form of the endogenous human complement regulator CR1 (CD35) with demonstrated ability to inhibit complement activation at the C3 and C5 convertases of the complement pathway, and demonstrated efficacy in numerous rat models of autoimmune and inflammatory diseases. The dose and frequency of sCR1 injection is based on the use of a similar treatment regimen with sCR1 in rats by Ramaglia et al. (2009) and Lillegard et al. (2013).

### Complement measurements

#### CH50

Total hemolytic complement activity was measured in serum obtained from tail vein or abdominal aorta. Briefly, the assay involves serial dilution of serum and incubation with antibody‐coated sheep red blood cells for 1 h at 37°C. The antibody‐coated cells are lysed by complement, releasing hemoglobin. The inverse of the dilution of serum resulting in lysis of 50% of the cells is reported as the CH50. CH50 is a general measure of the overall function of the classical pathway of complement activation.

#### C3a

The complement activation product C3a was measured by western blot as previously described (Lillegard et al. 2013). Modifications included the use of NuPAGE Novex 10% Bis‐Tris gels with MES SDS running buffer for separation of serum. Immunodetection used a custom primary rabbit polyclonal antibody to the C terminus of rat C3a and the secondary antibody IRDye 800CW Goat anti‐Rabbit IgG (H+L) at 1/10,000 dilution and LiCor Odyssey Fc for imaging. Rat serum was activated by incubation with yeast to generate a standard, with a standard curve included on each gel. Relative amounts of C3a were expressed as C3a units/*μ*L based on signal intensity of 1 *μ*L of standard pool of rat serum activated by yeast.

#### Complement regulators

Message for endogenous complement regulators in kidney and aorta of rats on low salt (0.4%) versus high salt (4.0%) diet was determined. Message for *Crry* and *Cd59* was assessed as previously described (Regal et al. 2016) using qRT PCR. The Delta‐delta Ct method of relative quantification was used to determine fold change in mRNA expression compared to actin with value for animals on low‐salt diet (0.4%) defined as 1.

### Statistical analysis

Data are expressed as the mean ± one standard error. Blood pressure and urine albumin excretion data were assessed for significance using a two‐way repeated measures analysis of variance (ANOVA) with a Holm‐Sidak post hoc test. Differences in CH50, C3a and fold changes in message for *Crry* and *Cd59* obtained from high‐salt animals treated with saline vehicle or sCR1 were assessed by Student's *t* test. A probability value of *P* < 0.05 was considered significant.

## Results

### Effect of complement inhibition on proteinuria and hypertension

**Figure 1:**
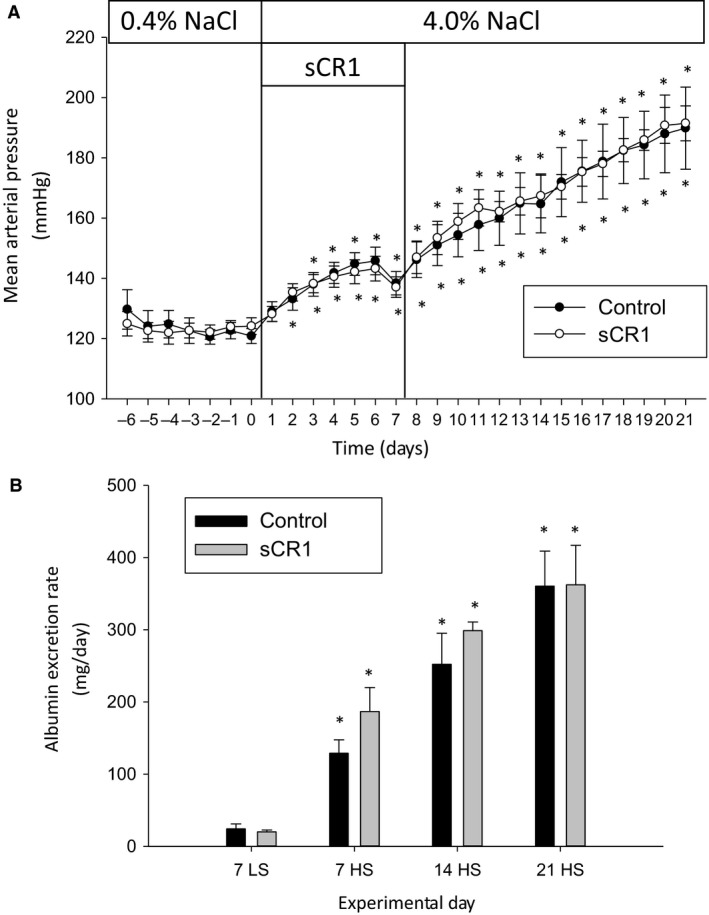
sCR1 administered days 1–7 of a high‐salt diet does not inhibit hypertension or albuminuria in Dahl SS rats. sCR1 or saline vehicle (Control) was administered ip at 15 mg/kg from day 1 when rats were changed from 0.4% (LS) low salt to 4.0% (HS) high‐salt diet until day 7, and the effect on telemetric blood pressure readings determined in 5–6 animals/treatment group. (A) Mean arterial blood pressure (MAP). (B) Urine albumin excretion rate. Overnight urine was collected prior to switching to high salt (7LS) and at 7, 14, and 21 days after high salt (7HS, 14HS, 21HS). *indicates *P* < 0.05 versus day 0 for MAP and versus LS7 for albumin excretion rate.

As depicted in Figure 1, the administration of a high salt (4.0% NaCl) diet to SS rats led to a significant increase in the 24 h average mean arterial blood pressure (MAP) and in albumin excretion rate in SS rats. sCR1 treatment from days 1 to 7 of high salt did not significantly alter the progression of the increase in MAP or albuminuria in the experimental group of animals (Fig. 1). Similar to the change in albumin excretion rate, protein excretion rate significantly increased from 61 ± 11 to 479 ± 48 mg/day in the control SS rats; treatment with sCR1 did not significantly alter the development of proteinuria in the experimental rats with excretion values not significantly different from control in any urine collection period. The sodium and potassium excretion rate were also not significantly different between the control and experimental groups during the low salt or the 21 day high salt urine collection period (Table 1). In the control group, sodium excretion rate significantly increased from 0.6 ± 0.2 mEq/day on the final day of the low‐salt diet to 13.3 ± 1.4 mEq/day after 21 days of the high‐salt diet; in contrast, potassium excretion was not significantly altered from the low‐salt diet excretion rate of 1.2 ± 0.1 mEq/day when compared to day 21 of the high‐salt diet in the control animals.

**Table 1:** Effect of sCR1 on urinary protein, sodium, and potassium excretion before high salt (7 LS) and 7, 14, or 21 days after high salt (7 HS, 14 HS, 21 HS)

	Protein excretion (mg/day)		Sodium excretion (mEq/day)		Potassium excretion (mEq/day)	
	Control	sCR1	Control	sCR1	Control	sCR1
sCR1 administered days 1–7 (*n* = 6 control, *n* = 5 sCR1)						
7 LS	61 ± 11	51 ± 5	0.6 ± 0.2	0.6 ± 0.2	1.2 ± 0.1	1.2 ± 0.1
7 HS	236 ± 28^a^	296 ± 49^a^	14.9 ± 1.4^a^	16.5 ± 0.9^a^	1.7 ± 0.1^a^	1.8 ± 0.1^a^
14 HS	377 ± 53^a^	417 ± 18^a^	15.3 ± 0.8^a^	12.3 ± 1.7^a^	1.4 ± 0.1	1.1 ± 0.1
21 HS	479 ± 48^a^	499 ± 55^a^	13.3 ± 1.4^a^	10.4 ± 1.6^a^	1.3 ± 0.1	1.2 ± 0.1
sCR1 administered days 14–18 (*n* = 7 control, *n* = 5 sCR1)						
7 LS	66 ± 12	52 ± 10	1.0 ± 0.1	0.6 ± 0.1	1.4 ± 0.1	1.2 ± 0.1
7 HS	213 ± 34^a^	191 ± 60^a^	15.3 ± 0.4^a^	13.6 ± 2.3^a^	1.6 ± 0.0^a^, ^b^	1.8 ± 0.1^a^
14 HS	303 ± 46^a^	326 ± 45^a^	14.2 ± 1.4^a^	14.2 ± 2.1^a^	1.4 ± 0.1	1.4 ± 0.1
21 HS	352 ± 35^a^	345 ± 51^a^	15.1 ± 0.9^a^	14.6 ± 1.6^a^	1.4 ± 0.0	1.4 ± 0.1

a *P* < 0.05 versus 7 LS, same group.

b *P* < 0.05 versus sCR1 treated at the same time point.

Serum was obtained from the tail vein of animals on day 8, one day after completion of the sCR1 treatment. As seen in Figure 3A, sCR1 treatment substantially and significantly decreased CH50 when measured from serum collected on day 8, indicating adequate inhibitory concentrations were achieved for 24 h following ip injection. CH50 in serum collected on day 21 from these animals had returned to normal values by time of euthanasia on day 21. When compared to control, sCR1 also significantly decreased serum C3a when measured in serum on day 8, 24 h after the last ip injection of sCR1 (Fig. 3B).

**Figure 2:**
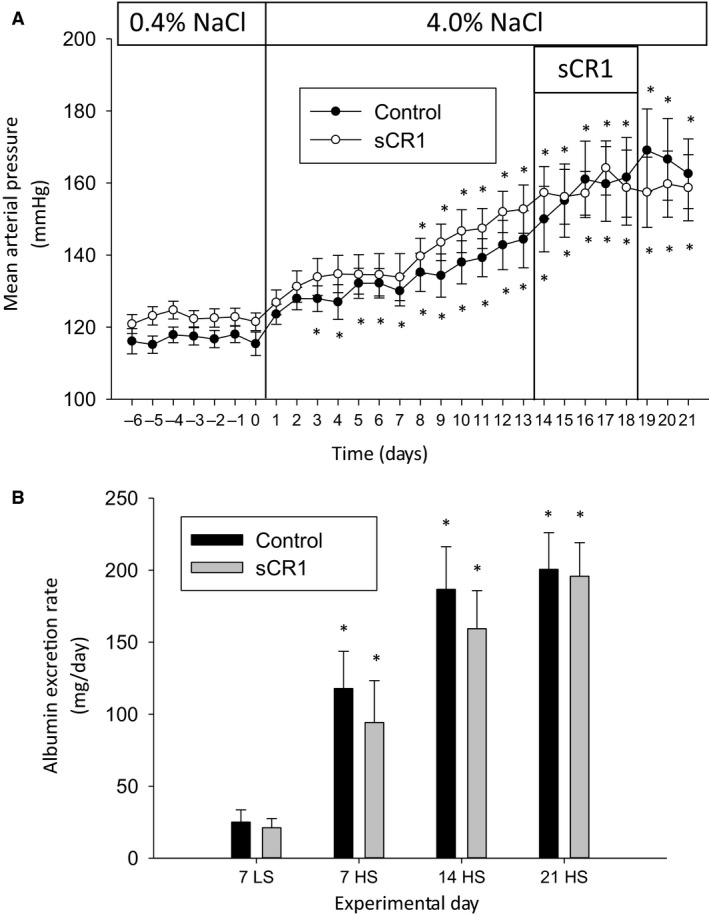
sCR1 administered days 14–18 of a high‐salt diet does not inhibit hypertension or albuminuria in Dahl SS rats. sCR1 or saline vehicle (Control) was administered ip at 15 mg/kg days 14–18 and the effect on telemetric blood pressure readings determined in 5–6 animals/treatment group. 0.4% (LS) low salt, 4.0% (HS) high salt. (A) Mean arterial blood pressure (MAP). (B) Urine albumin excretion rate. Overnight urine was collected prior to switching to high salt (7LS) and at 7, 14, and 21 days after high salt (7HS, 14HS, 21HS). * indicates *P* < 0.05 versus day 0 for MAP and versus LS7 for albumin excretion rate.

A second group of animals were treated with sCR1 or saline vehicle on days 14–18 of the high‐salt diet as seen in Figure 2. Again, sCR1 treatment did not significantly alter the progression of the increase in MAP or albuminuria in the SS rats fed the high‐salt diet. Similar to the results described for group 1, protein excretion rate tracked with albumin excretion rate (increased from 66 ± 12 to 352 ± 35 mg/day in the control SS rats) and was not significantly different between the control and sCR1‐treated groups. The sodium and potassium excretion rate were also not significantly different between the control and experimental groups during the low salt or the 21 day high salt urine collection period (Table 1). In the control group, sodium excretion rate significantly increased from 1.0 ± 0.1 mEq/day on the final day of the low‐salt diet to 15.1 ± 0.9 mEq/day after 21 days of the high salt diet; in contrast, potassium excretion was not significantly altered from the low‐salt diet excretion rate of 1.4 ± 0.1 mEq/day when compared to day 21 of the high‐salt diet in the control animals.

**Figure 3:**
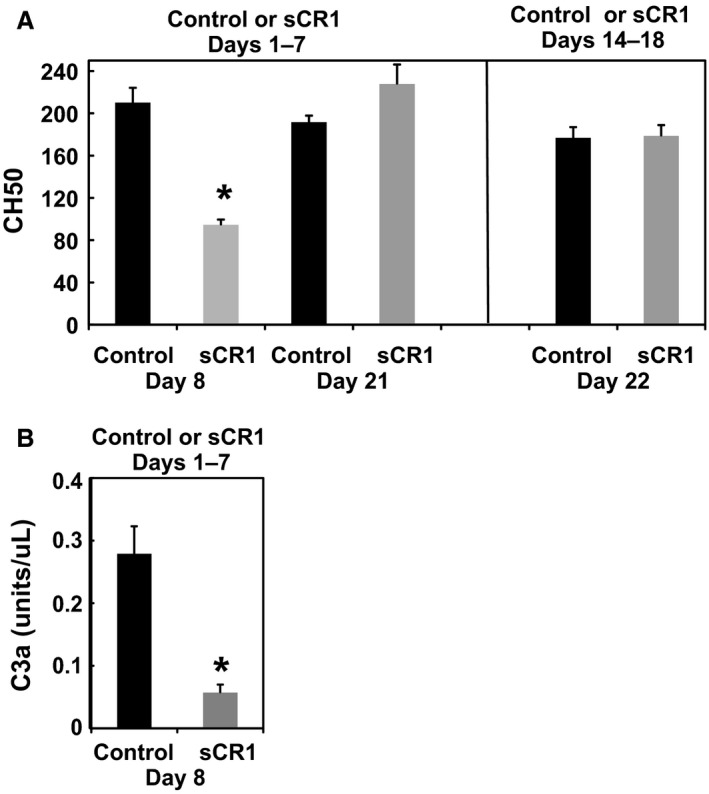
(A) Effect of saline vehicle (Control) or sCR1 on total hemolytic complement activity (CH50) in the Dahl SS rat in animals treated either Days 1–7 (Figure 1) or Days 14–18 (Figure 2). CH50 was determined in serum obtained on day 8 and Day 21 in animals treated in the initial phase of high salt, or on day 22 in animals treated in the later phase of the hypertensive response. (B) Effect of saline vehicle (Control) or sCR1 on C3a in the Dahl SS rat in animals treated Days 1–7 (Figure 1). C3a was determined by western blot in serum collected on day 8. *P < 0.05 versus Control by Student's *t* test.

CH50 was measured 4 days after cessation of sCR1 treatment in this group of animals. As seen in Figure 3A, the CH50 had returned to normal 4 days after the last sCR1 injection (day 22).

### Effect of high‐salt diet on endogenous complement regulators

**Figure 4:**
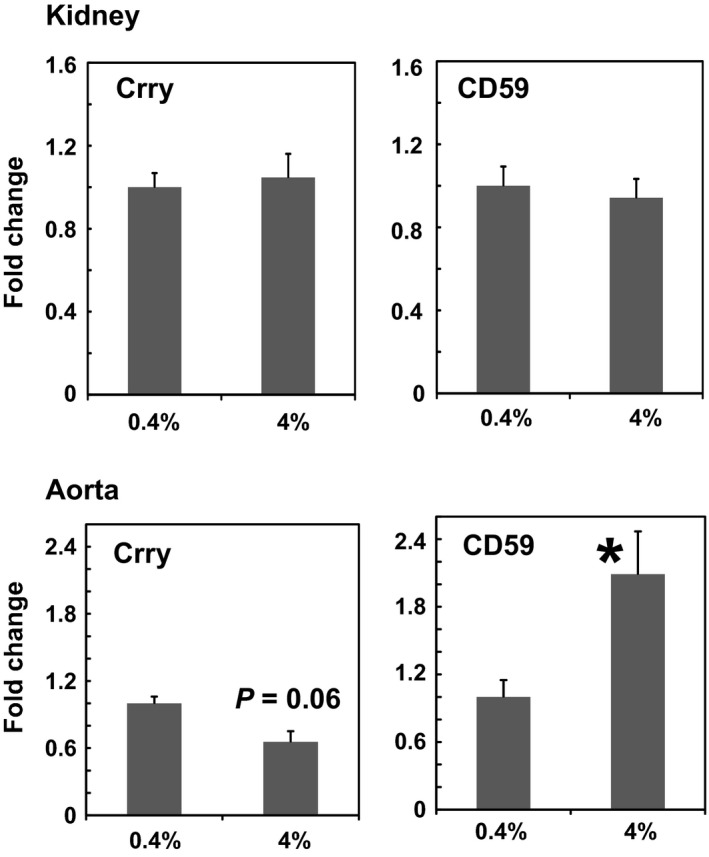
Fold change in message for complement regulators *Crry* and *Cd59* in kidney cortex or aorta obtained from SS rats on either low salt (0.4%) or high salt (4%) diet for 21 days. Values represent the mean+SE of 3 animals/treatment group. The Delta‐delta Ct method of relative quantification was used to determine fold change in mRNA expression compared to actin with change in animals on low‐salt diet (0.4%) defined as 1. *P < 0.05 versus 0.4% treatment group by Student's *t* test.

Mammalian cells have numerous endogenous molecules that control complement activation to prevent deleterious effects of C3 deposition and formation of the membrane attack complex on our own cells. This includes the endogenous membrane proteins complement receptor 1‐related protein/gene y (CRRY) and CD59. CRRY is a rodent protein with activities of both human DAF (CD55) and MCP (CD46). CRRY primarily controls activation at the C3/C5 convertase and C3 deposition by accelerating decay of the active enzyme as well as acting as a cofactor for inactivation of C3b and C4b, components of both the alternative and classical pathway convertases. CD59 prevents C9 from associating with C5b‐8, and thus operates to prevent formation of the terminal lytic pathway of complement activation, C5b‐9 (membrane attack complex). We hypothesized that high salt resulted in a reduction in complement regulators in the kidney cortex or vasculature resulting in increased C3 convertase activity and complement activation that overwhelmed the inhibitory capacity of the exogenous sCR1 administered. sCR1 also inhibits the C3/C5 convertase via decay acceleration of the enzyme as well as having cofactor activity for inactivation of C3b and C4b. As seen in kidney cortex (Fig. 4) the message for *Crry* and *Cd59* was not affected by high‐salt diet. If message for the same regulators was assessed in aorta, high salt tended to decrease *Crry* in aorta (*P* = 0.06) and significantly increased *Cd59* (*P* < 0.05). Thus, high salt caused minor decreases or no significant change in *Crry* that controls C3 convertase, with a large increase in *Cd59* that controls formation of C5b‐9. Overall, the small decrease in *Crry* controlling the C3 convertase is not likely responsible for the ineffectiveness of sCR1 in altering hypertension or albuminuria with a high‐salt diet.

## Discussion

Excess complement system activation has been implicated in pathophysiology of numerous types of hypertension and kidney disease. Two recent transcriptomic studies had indicated that the complement pathway is upregulated in the kidney of the Dahl SS rat fed a high‐salt diet (Liu et al. 2014; Geurts et al. 2015), and thus we hypothesized that the hypertension and renal disease induced by high salt may in part be due to excessive complement activation. We utilized the recombinant molecule sCR1 to inhibit complement activation in the Dahl SS rat and determine if complement activation was critical for either the early or late phase of the development of the hypertension and proteinuria induced by high salt. Administration of sCR1 either immediately after the high‐salt diet was initiated or in the later phases of the development of severe hypertension and proteinuria did not affect progression of the disease phenotype. Thus, complement activation in the circulation is clearly not critical for development of salt sensitive hypertension and proteinuria in this animal model.

As we have demonstrated, pharmacological or genetic blockade of the immune system does not prevent hypertension and related kidney damage in the Dahl SS rat, but attenuates the disease process (Mattson et al. 2006, 2013). The hypertension and proteinuria develop in phases with neoantigen formation, infiltrating immune cells, and reactive oxygen species leading to renal vasoconstriction and blunted pressure natriuresis (Mattson 2014; Evans et al. 2015). Mediators such as IL‐6 contribute to hypertension and proteinuria primarily in the late phase of hypertension development (Hashmat et al. 2016). Our data indicates that the hemolytic complement activity was significantly reduced in the circulation 24 h after sCR1 treatment on day 8 following high‐salt diet in the early phase. However, we did not collect an analogous sample 24 h after sCR1 treatment in the later phase of the hypertensive response on day 19 to conclusively indicate that this dose of sCR1 was sufficient to inhibit complement activation in the circulation in the later phase with extensive injury and severe salt sensitive hypertension. Our results do not provide supportive evidence that complement system activation is critical for development of either the early or late phase of hypertension or albuminuria in the Dahl SS rat, suggesting that upregulation of the complement system in the kidney is a response to the injury.

sCR1 has been used in numerous animal models of autoimmune and inflammatory disease. It is a recombinant human molecule that has been demonstrated to be effective in inhibiting complement activation in the rat, mouse, and guinea pig (Regal et al. 1993; Lillegard et al. 2013). A limitation of using human sCR1 in rodent models is that only short‐term treatment is possible because of the inevitable development of an immune response to the molecule (Ruggieri et al. 1996). The dose used in this study was based on a variety of early studies using 10–20 mg/kg of this molecule to inhibit complement activation in the rat or pig (Piddlesden et al. 1994, 1996; Goodfellow et al. 1997; Chai et al. 2000), as well as our more recent study in the pregnant rat where the hypertension induced by reduced placental perfusion was significantly attenuated by daily administration of 15 mg/kg sCR1 (Lillegard et al. 2013) over a 4–5 day time period. In addition, increasing the dose to 30 mg/kg did not have any greater effect on placental ischemia‐induced hypertension than 15 mg/kg (Lillegard et al. 2013). Our study of placental ischemia‐induced hypertension was the first study to link complement activation to hypertension. The reduction in CH50 in the Dahl SS rat was similar to that achieved in our study in the pregnant rat (Lillegard et al. 2013) indicating that the dose was inhibiting complement activation in the circulation in the Dahl SS rat as expected. More recent studies in the wildtype PVG rat demonstrated 15 mg/kg per day sCR1 resulted in 80–90% inhibition of CH50 (Ramaglia et al. 2009) with sCR1 favorably altering the course of peripheral nerve injury. In the mouse 25 mg/kg sCR1 is effective at reducing C3 deposition in kidney in a model of C3 glomerulopathy (Zhang et al. 2013). Original studies with sCR1 in glomerulosclerosis reported that 60 mg/kg for 2–3 days had a positive effect in mouse models of acute glomerulosclerosis known to be complement dependent. Certainly, higher or more prolonged dosing with sCR1 or administration prior to rather than coincident with the change to high salt may have been more effective in inhibiting complement activation in kidney and other tissue sites and thus altering the phenotype of salt sensitive hypertension in our study. However, no indication of any change in hypertension or albuminuria was noted in our studies to prompt further investigations at higher doses of sCR1.

It is not known if high salt results in complement activation and C3 deposition in the kidney of the Dahl SS rat and this was not an endpoint measured in this study. A clear limitation of our study is the possibility that the important site of inhibition of complement activation may be locally in the tissues and the effectiveness of sCR1 at this tissue site is unknown. It is possible that stronger complement inhibitors targeted to the site of complement activation may be effective in salt‐sensitive hypertension. CR2‐CRRY or Ig‐CRRY are complement inhibitors that have clearly improved kidney function in a mouse model of lupus nephritis (Bao et al. 2003; Atkinson et al. 2008). Numerous efforts are underway to develop therapeutically useful complement inhibitors in kidney disease (Ricklin et al. 2018). Our studies clearly demonstrate that sCR1 at 15 mg/kg per day markedly reduces complement activity in the circulation, but does not alter the salt‐sensitive phenotype suggesting that activation of complement in the circulation is not critical for the hypertension and proteinuria in this model.

Regulation of complement activation is essential to prevent excess complement activation from damaging our own cells. As with any enzymatic amplification system, the initiation and activation of the complement cascade is balanced by endogenous regulators that curb activation generally or locally. CRRY is one of the endogenous regulators that controls complement activation at C3 to prevent excessive cleavage of the C3 molecule into C3a and C3b. CD59 on the other hand prevents activation of the complement cascade at the C5b‐9 stage. If excessive complement activation occurred in response to high salt, and the endogenous regulators were simultaneously decreased, then addition of exogenous sCR1 may be insufficient to contain and control the complement system. However, minimal decreases in message for the complement regulators were evident at the C3 convertase level in the kidney, and *Cd59* in the aorta was upregulated suggesting that activation of the terminal complement pathway was controlled. Our study is limited in that many other endogenous regulators of complement activation may influence the level of complement activation and were not assessed in the present study (Regal et al. 2017).

Overall, this study indicates that complement activation is not critical for development of the salt‐sensitive phenotype, though changes in the complement system may be occurring. Thus, the immune cell infiltration that amplifies the disease phenotype and plays a critical role in the development of hypertension and kidney damage is not dependent on the innate immune complement system.

## Conflicts of Interest

None declared.
